# Molecular Epidemiology and Evolution of Influenza Viruses Circulating within European Swine between 2009 and 2013

**DOI:** 10.1128/JVI.00840-15

**Published:** 2015-07-22

**Authors:** Simon J. Watson, Pinky Langat, Scott M. Reid, Tommy Tsan-Yuk Lam, Matthew Cotten, Michael Kelly, Kristien Van Reeth, Yu Qiu, Gaëlle Simon, Emilie Bonin, Emanuela Foni, Chiara Chiapponi, Lars Larsen, Charlotte Hjulsager, Iwona Markowska-Daniel, Kinga Urbaniak, Ralf Dürrwald, Michael Schlegel, Anita Huovilainen, Irit Davidson, Ádám Dán, Willie Loeffen, Stephanie Edwards, Michel Bublot, Thais Vila, Jaime Maldonado, Laura Valls, ESNIP3 Consortium, Ian H. Brown, Oliver G. Pybus, Paul Kellam

**Affiliations:** a Wellcome Trust Sanger Institute, Hinxton, United Kingdom; b Animal and Plant Health Agency, Addlestone, Surrey, United Kingdom; c Department of Zoology, University of Oxford, Oxford, United Kingdom; d Laboratory of Virology, Ghent University, Merelbeke, Belgium; e Anses, Ploufragan-Plouzané Laboratory, Swine Virology Immunology Unit, Ploufragan, France; f Istituto Zooprofilattico Sperimentale della Lombardia e dell'Emilia Romagna, Parma, Italy; g Department of Veterinary Diagnostics and Research, Technical University of Denmark, Copenhagen, Denmark; h Department of Swine Diseases, Panstwowy Instytut Weterynaryjny, Pulawy, Poland; i IDT Biologika GmbH, Dessau-Roßlau, Germany; j Finnish Food Safety Authority EVIRA, Helsinki, Finland; k Division of Avian Diseases, Kimron Veterinary Institute, Bet Dagan, Israel; l National Food Chain Safety Office, Budapest, Hungary; m Central Veterinary Institute, Wageningen UR, Lelystad, The Netherlands; n Virology Department, Discovery Research, Merial, Lyon, France; o Veterinary Diagnostic Services DIAGNOS, Laboratorios HIPRA SA, Gerona, Spain; p Division of Infection & Immunity, University College London, London, United Kingdom

## Abstract

The emergence in humans of the A(H1N1)pdm09 influenza virus, a complex reassortant virus of swine origin, highlighted the importance of worldwide influenza virus surveillance in swine. To date, large-scale surveillance studies have been reported for southern China and North America, but such data have not yet been described for Europe. We report the first large-scale genomic characterization of 290 swine influenza viruses collected from 14 European countries between 2009 and 2013. A total of 23 distinct genotypes were identified, with the 7 most common comprising 82% of the incidence. Contrasting epidemiological dynamics were observed for two of these genotypes, H1_hu_N2 and H3N2, with the former showing multiple long-lived geographically isolated lineages, while the latter had short-lived geographically diffuse lineages. At least 32 human-swine transmission events have resulted in A(H1N1)pdm09 becoming established at a mean frequency of 8% across European countries. Notably, swine in the United Kingdom have largely had a replacement of the endemic Eurasian avian virus-like (“avian-like”) genotypes with A(H1N1)pdm09-derived genotypes. The high number of reassortant genotypes observed in European swine, combined with the identification of a genotype similar to the A(H3N2)v genotype in North America, underlines the importance of continued swine surveillance in Europe for the purposes of maintaining public health. This report further reveals that the emergences and drivers of virus evolution in swine differ at the global level.

**IMPORTANCE** The influenza A(H1N1)pdm09 virus contains a reassortant genome with segments derived from separate virus lineages that evolved in different regions of the world. In particular, its neuraminidase and matrix segments were derived from the Eurasian avian virus-like (“avian-like”) lineage that emerged in European swine in the 1970s. However, while large-scale genomic characterization of swine has been reported for southern China and North America, no equivalent study has yet been reported for Europe. Surveillance of swine herds across Europe between 2009 and 2013 revealed that the A(H1N1)pdm09 virus is established in European swine, increasing the number of circulating lineages in the region and increasing the possibility of the emergence of a genotype with human pandemic potential. It also has implications for veterinary health, making prevention through vaccination more challenging. The identification of a genotype similar to the A(H3N2)v genotype, causing zoonoses at North American agricultural fairs, underlines the importance of continued genomic characterization in European swine.

## INTRODUCTION

Swine influenza viruses (swIAV) cause influenza in pigs, a disease that results in significant morbidity in swine herds across the world. swIAV outbreaks are due to infection with influenza A viruses (IAV), however, the genetic diversity of all circulating swIAV can be retraced using phylogenetic methods to avian progenitor viruses, often via humans (1). The first documented case of swIAV was in North America during the 1918 pandemic of human H1N1 influenza A virus (IAV) (2). This H1N1 subtype is thought to have transmitted from birds to humans shortly before 1918, with the human virus likely to have transferred into swine during the pandemic (3, 4). This virus became established in swine in the United States, forming the lineage now referred to as “classical swine H1N1” (CS) (1, 2, 4). Despite the prevalence of this lineage in North America and Asia, it did not become established in Europe until 1976, when infected pigs imported from the United States resulted in an outbreak in Italy that subsequently spread throughout European swine (1, 5).

European swine were infected solely by CS lineage viruses until 1979, when an avian H1N1 virus, genetically distinct from the CS lineage, was isolated from pigs in Belgium and Germany (1, 2, 6–8). This virus, now called “Eurasian avian-like swine H1N1” (EA), rapidly spread throughout Europe, outcompeting the preexisting CS viruses (1). The EA lineage continues to circulate among European swine and has reassorted with human seasonal-origin viruses since its emergence, resulting in the cocirculation of three distinct virus subtypes in Europe: (i) Eurasian avian-like H1_av_N1; (ii) A/swine/Gent/1/1984-like H3N2 (Gent/84); and (iii) A/swine/Scotland/410440/1994-like H1_hu_N2 (Scot/94) (2, 9–13).

In April 2009, a novel H1N1 IAV was isolated from humans in Mexico and the United States (14). This virus rapidly spread throughout the human population, causing the first global influenza pandemic of the 21st century. Studies showed that this virus, named A(H1N1)pdm09, was of swine origin and arose from the reassortment of an EA H1_av_N1 virus with a “triple-reassortant” (TR) swine virus that has circulated in North America and Asia since 1997 (15). The emergence of pandemic IAV from a swine source rather than an avian source was unexpected (16, 17). Furthermore, its complex reassortant history (involving swIAV circulating in separate regions of the world), combined with the length of time that the lineage had persisted without being detected, highlighted the need for more swIAV surveillance in swine worldwide (14). As a result, surveillance of swIAV has increased globally since 2009 (18–20), and large-scale whole-genome-sequencing studies have been reported for southern China and Hong Kong (21, 22) and for North America (23, 24). These studies discovered complex IAV diversity in swine, with high levels of reassortment between the enzootic lineages. Despite the importance of European-derived viruses in the genesis of the A(H1N1)pdm09 genotype, no large-scale whole-genome study has been reported for Europe. Therefore, the European Surveillance Network for Influenza in Pigs 3 (ESNIP3) was formed as an active swine surveillance network in participating European countries, representing the largest consortium for coordinated monitoring of IAV in pigs in Europe. As part of its work, the network undertook whole-genome sequencing of isolates from consortium partners sampled since the emergence of A(H1N1)pdm09 in 2009. Here we report the genomic diversity and molecular epidemiology of swIAV in Europe. Our report characterizes a total of 290 swIAV isolates collected from 14 countries between 2009 and 2013 and reveals significant genotypic diversity of swIAV in European swine for the first time, as well as substantial intracontinental differences in swIAV epidemiology.

## MATERIALS AND METHODS

### Surveillance and sample collection.

ESNIP3 consortium partners carried out influenza surveillance between 2010 and 2013 on swine farms with outbreaks of respiratory disease. Preliminary subtyping was performed on the isolated viruses at the time of sample collection (25). Samples positive for swIAV were selected for further antigenic and genetic characterization based on their geographic location, date of collection, and viral subtype to capture the diversity of the circulating viruses across Europe. Further details of the total numbers of swIAV detected in each country, and thus the proportion that were sent for sequencing, can be found in reference 25. All selected samples for each country were sent to a central virus bank at the UK Animal and Plant Health Agency, where they were cultured in embryonated fowls' eggs prior to viral RNA extraction.

### PCR amplification and virus sequencing.

Viral RNA amplification was performed using an 8-segment reverse-transcription-PCR (RT-PCR) procedure as previously described (26) and the modified primers described by Baillie et al. (27). Amplicons for each sample were pooled and then individually indexed and processed into libraries either through the use of standard Roche Rapid Library Prep for the 454 sequencing platform or as described by Quail et al. (2008) for the Illumina platform (28). Isolates were either sequenced on a Genome Sequencer FLX Titanium XL+ instrument (Roche/454 Life Sciences) or sequenced on a MiSeq instrument (Illumina) using a 150-bp paired-end reagent kit.

### Genome assembly.

Data generated by either platform were subjected to quality control using QUASR version 7.01 to remove any primer sequences and trim reads by applying a median-read-quality cutoff, as previously described (29). Quality-controlled read sets were *de novo* assembled using IVA version 0.8.1 (30), and custom Python scripts were used to remove any assembled contiguous sequences (“contigs”) that either were not of influenza origin or did not contain at least 70% of the expected open reading frame length for that segment. In addition, read sets were assembled against a reference sequence using SMALT version 0.7.4 (www.sanger.ac.uk/resources/software/smalt/), with the reference selected using custom Python scripts that perform a BLAST search on a subset of the reads and download the most frequent hit for each segment. Output files generated by SMALT were parsed using SAMtools version 0.1.8 (31) and QUASR to generate consensus sequences. These consensus sequences were used to fill in segments unable to be assembled by the *de novo* assembler. Python scripts are available from us on request.

For samples where multiple contigs could be generated for one or more segments, the lineage of origin for all segments (i.e., classical swine, Eurasian avian-like, 2009 pandemic, human seasonal-derived, “triple reassortant,” or avian) was determined. Where samples had one or two segments with contigs of different lineages, the contig whose lineage matched the remaining segments was selected, with the others considered contaminants and removed. Where multiple genotypes could be constructed (e.g., complete pdm09 and EA genotypes in a sample), a custom Python script was used to calculate the relative abundance of each genotype in the original reads. If the genotype was present at <5% in the sample, it was considered a contaminant and discarded. Using this stringent quality check, no mixed infections were detected in any of the samples.

### Phylogenetic analysis.

The influenza virus sequences were combined with existing sequences retrieved from the NCBI Influenza Virus Resource (32) that represented the range of genetic diversity of swIAV worldwide. All available European swine isolates were included. Each genome segment was aligned separately using the MUSCLE aligner (33) provided in MEGA version 6.06 (34). Separate alignments were made for H1, H3, N1, and N2 sequences. Alignments were then trimmed to coding regions, and sequences covering less than 50% of the coding region were removed. The resultant data sets contained between 763 (N1) and 2,405 (H3) sequences. Phylogenetic trees were then inferred under a maximum-likelihood (ML) criterion using RAxML version 7.2.8 (35). Phylogenies were inferred under the general time-reversible nucleotide substitution model, with the rate of heterogeneity among sites modeled as a 4-category discrete gamma distribution (GTR+Γ_4_). Tree robustness was determined through bootstrap analysis of 1,000 sequence pseudoreplicates. Trees were visualized using FigTree version 1.4.2 (http://tree.bio.ed.ac.uk/software/figtree/).

### Genotype assignment.

From the ML phylogenies, virus isolates were categorized into lineages circulating in swine worldwide, with a particular emphasis on Europe, as follows: (i) Eurasian avian-like H1_av_N1; (ii) A/swine/Gent/1/1984-like H3N2; (iii) A/swine/Scotland/410440/1994-like H1_hu_N2; (iv) A/swine/Italy/4675/2003-like rH1N2; (v) North American triple reassortant; (vi) classical H1N1; (vii) A(H1N1)pdm09; (viii) human seasonal H3N2; and (ix) avian. Each segment for a sample was assigned to one of the nine lineages defined above to generate a complete genotype for that sample. For the purposes of genotype classification, where samples had one or more missing internal gene segments (PB2, PB1, PA, NP, MP, or NS), the lineage of the missing segments was assigned to those of the sequenced internal genes. This assumption is reasonable because of the negligible rate of reassortment observed within the internal gene cassette (IGC) of the other isolates in this study. Where no internal gene segments were obtained, the genotype was specified as “undetermined.” Furthermore, if either of the external glycoprotein segments (hemagglutinin [HA] or neuraminidase [NA]) were missing, the genotype was also considered undetermined.

Isolates collected by the ESNIP3 project that had already been deposited in GenBank were removed to avoid duplication of isolates. Furthermore, to avoid the misrepresentation of genotype proportions across Europe, only a single isolate representative of an outbreak was retained per country per year. The ML phylogenies for all segments were scrutinized, and where viruses grouped together in a well-supported clade across all eight segments, they were considered to have originated in the same outbreak. A single sample was chosen to represent that outbreak per year, retaining the ESNIP3 isolate where appropriate.

### Lineage-specific phylogenies.

Molecular phylogenies were estimated for the H1 and N2 genes for the Scot/94 lineage and for the N2 gene for the Gent/84 lineage. These were inferred using MrBayes version 3.2.2 (36, 37), with three Bayesian Markov Chain Monte Carlo (BMCMC) chains run for 2 million states under a GTR+Γ_4_ substitution model. A 25% burn-in was discarded after assessing convergence using Tracer version 1.6 (http://tree.bio.ed.ac.uk/software/tracer/).

### Molecular clock phylogeny.

A time-resolved phylogeny was estimated for the A(H1N1)pdm09 lineage in order to investigate the transmission of this virus from humans to swine. All European human influenza A(H1N1)pdm09 IGC sequences were downloaded from the NCBI Influenza Virus Resource, along with their collection dates. After each segment was separately aligned and trimmed to its coding region using MEGA version 6.06, the segments were concatenated. A Python script was used to downsample the sequences to remove highly similar sequences sampled from the same geographic location and month. These were then combined with the previously curated A(H1N1)pdm09 swine IGC sequences. Molecular clock Bayesian phylogenies were inferred using BEAST version 1.8.0 (38, 39), under a GTR+ Γ_4_ substitution model. A strict molecular clock was used after assessing the clock-likeness of the data using Path-O-Gen version 1.4 (http://tree.bio.ed.ac.uk/software/pathogen/), and a Bayesian Skyride coalescent model was used to model demographic history (40). Three BMCMC chains were run for 100 million states, with samples taken every 10,000 states. A 10% burn-in was used, and convergence of the chains was assessed using Tracer version 1.6.

### Statistical analyses.

The null hypothesis of no association between the host species and the phylogenetic ancestry of the A(H1N1)pdm09 lineage was tested using Bayesian Tip-association Significance Testing (BaTS) beta build 2 (41). All full-length human and swine A(H1N1)pdm09 PB2, HA-H1, and NA-N1 sequences were downloaded from the NCBI Influenza Virus Resource and combined with the A(H1N1)pdm09 swine sequences generated in this study. Separate alignments were made for each segment, and alignments were trimmed to the coding region. Bayesian posterior sets of trees were inferred using MrBayes version 3.2.2 as described above. Custom Python scripts were written to generate the BaTS input Nexus file where each taxon is labeled with its corresponding host species.

### Nucleotide sequence accession numbers.

The generated nucleotide sequences are available in GenBank under accession numbers KR699644 to KR701609 .

## RESULTS

### Genotypic diversity.

A total of 243 viruses, provided by the ESNIP3 consortium, that circulated between 2009 and 2013 were used to generate 231 complete and 12 incomplete genomes using high-throughput sequencing platforms. These were combined with the 47 genomes present in GenBank to generate a set of 290 swIAV genomes from 14 countries across Europe: Belgium, Denmark, Finland, France, Germany, Hungary, Israel, Italy, The Netherlands, Norway, Poland, Spain, Sweden, and the United Kingdom (see Table S1 in the supplemental material).

**FIG 1:**
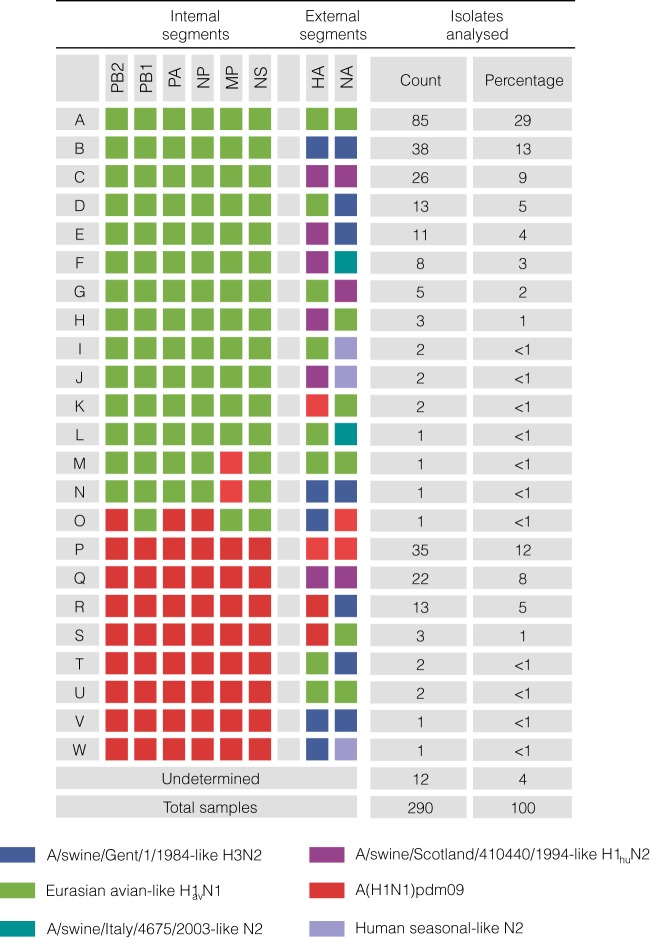
swIAV genotypes isolated from European swine between 2009 and 2013. The 23 distinct genetic constellations are labeled A to W, with the lineage of origin for each segment indicated by a colored block.

From these data, 23 different genotypes (A to W) were observed across Europe (Fig. 1). 12 genotypes (A to L), comprising 67% of the isolates, contained an internal gene cassette (IGC) derived from the Eurasian avian-like (EA) lineage, while eight genotypes (P to W), comprising 27% of the isolates, contained an IGC derived from the A(H1N1)pdm09 lineage. Three genotypes (M, N, and O), each observed only once, contained a reassortant IGC with segments from both the EA and A(H1N1)pdm09 lineages. The majority of the observed genetic diversity was therefore generated through reassortment of the glycoprotein-encoding segments of the four major lineages circulating in European swine: (i) EA H1_av_N1; (ii) A/swine/Scotland/410440/1994-like H1_hu_N2; (iii) A/swine/Gent/1/1984-like H3N2; and (iv) A(H1N1)pdm09. Additional diversity in the neuraminidase (NA) segment was provided through the circulation of the A/swine/Italy/4675/2003 “human-like” N2 lineage and sporadic reverse zoonoses of human H3N2 seasonal viruses.

Despite 23 distinct swIAV genotypes being identified in European swine, 82% of the samples could be attributed to the seven most frequent genotypes (A, B, C, D, P, Q, and R). The remaining 18% represented rarer and geographically constrained reassortant genotypes (Fig. 1).

### Frequency of EA-based genotypes across Europe.

**FIG 2:**
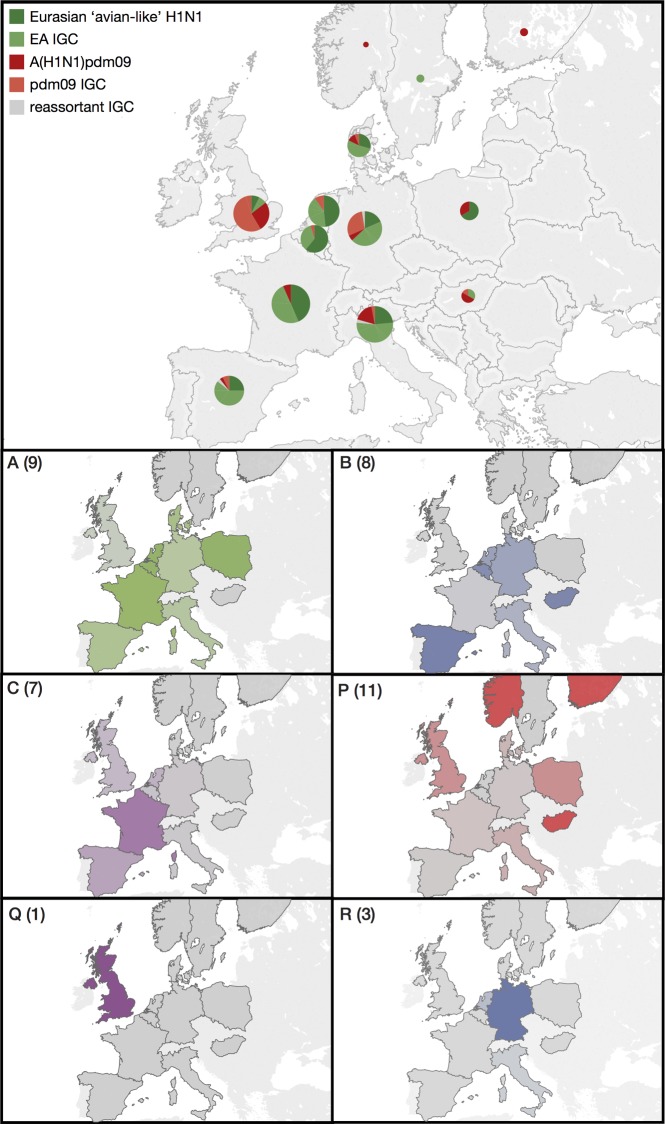
Frequencies of the swIAV genotypes across Europe. (Upper panel) Pie charts indicate the proportions of samples isolated in each country that were either EA H1N1 (dark green) or A(H1N1)pdm09 (dark red) or contained either an EA IGC (light green) or a pdm09 IGC (light red). Isolates whose IGC contained both EA and pdm09 segments are colored gray. The sizes of the pie charts reflect the number of samples used for analysis from each country as follows: Belgium, 24; Denmark, 17; Finland, 2; France, 47; Germany, 38; Hungary, 6; Italy, 42; The Netherlands, 30; Norway, 1; Poland, 11; Spain, 28; Sweden, 2; United Kingdom, 41. For clarity, the data for Israel [*n* = 1; A(H1N1)pdm09 (pdm09)] are not shown. IGC, internal gene cassette. (Panels A to R) Relative frequencies of the six most prevalent swIAV genotypes in countries across Europe. Each genotype is specified as described for Fig. 1. The intensity of the color in each panel reflects the relative frequency of the genotype in that country. Countries that provided no samples are not outlined. Numbers in parentheses indicate the number of countries each genotype was isolated from. Maps were created using Tableau version 9.0.0.

The proportions of each circulating swIAV genotype differed among European regions (Fig. 2). This was most starkly revealed in the contrast between the United Kingdom and mainland Europe. Swine in mainland Europe were predominantly infected by EA-based genotypes (A to L); among countries where more than 10 isolates were analyzed, the mean percentage of all these genotypes together was 83% ± 11%. United Kingdom swine, conversely, were predominantly infected by A(H1N1)pdm09-based genotypes (P to W), with the proportion of the EA-based genotypes being only 15%.

Other geographic trends within mainland Europe were less pronounced and possibly also reflected different sampling biases (Fig. 2). EA H1_av_N1 (genotype A) was found across mainland Europe at an average prevalence of 37% ± 16%; the frequency was highest in Belgium (58%) and lowest in Germany (18%) (Fig. 2A). Gent/84-like H3N2 (genotype B) was observed across mainland Europe at an average frequency of 15% ± 14%, with Spain (36%) and Hungary (33%; *n* = 6) having the highest frequencies, but only a single outbreak of this genotype was isolated in France, close to the Belgian border. This genotype was not isolated in Denmark, Poland, or the United Kingdom (Fig. 2B). The Scot/94-like H1_hu_N2 genotype (genotype C) was present across mainland Europe at an average prevalence of 7% ± 10% and at 7% in the United Kingdom. The prevalence of this genotype appears to be inversely proportional to that of genotype B, having a higher relative frequency in countries where genotype B had a low frequency and vice versa (Fig. 2C). Its highest frequency was in France (30%), while Belgium, Germany, and Italy had only a single isolate, and the genotype was not isolated in Denmark. Instead, Danish swine were predominantly infected with a reassortant rH1_av_N2 (genotype D). This was the most frequent genotype in Denmark (47%), but only two isolates of genotype D were identified in Sweden, and single isolates were observed in The Netherlands, Germany and Italy (see Fig. S1 in the supplemental material).

The remaining EA-derived genotypes (E to L) were geographically constrained reassortants found either at a significant percentage in one country with sporadic occurrences in a neighboring country (genotypes E and G) or as transient isolated occurrences within a country (genotypes H to L). A genotype of particular note is genotype F, which contains EA-derived internal genes and a Scot/94-derived HA but whose NA originated from a human-to-swine transmission separate from the Gent/84 and Scot/94 transmission events. This was found circulating at 19% prevalence in Italy, where it originally arose (42).

### Frequency of A(H1N1)pdm09 genotypes across Europe.

Following the outbreak of the human pandemic in April 2009, A(H1N1)pdm09 influenza (genotype P) was detected in European swine as early as September 2009 (43). Since then, the pandemic virus has been isolated from swine in countries across Europe at various frequencies, with those isolated in United Kingdom and Poland each having the greatest frequency at 27%, while those isolated in mainland Europe on average had a frequency of 8% ± 9% in countries with more than 10 samples (Fig. 2). The pandemic virus was not detected in swine in Belgium or The Netherlands.

**FIG 3:**
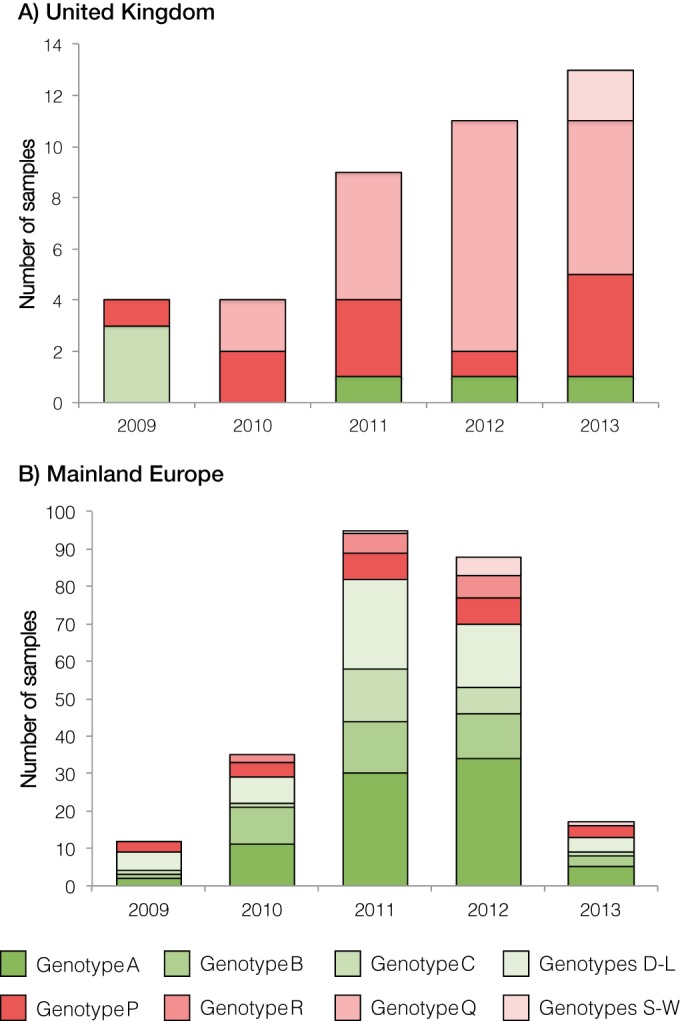
Comparison of swIAV genotypes isolated in the United Kingdom (A) and mainland Europe (B). Bar charts are colored according to the corresponding genotypes, with genotypes specified as given in Fig. 1. The six most prevalent genotypes are shown separately, with the remaining genotypes clustered according to the lineage of the corresponding internal gene cassettes.

Following the introduction of A(H1N1)pdm09 into European swine, reassortants between this genotype and the established genotypes circulating within Europe soon emerged. In the United Kingdom, the pandemic virus reassorted with enzootic Scot/94-derived H1_hu_N2, acquiring the external glycoproteins (genotype Q) (Fig. 2). This genotype was first observed in 2010 and rapidly replaced genotype C; from 2010 to 2013, no genotype C was isolated in the United Kingdom (Fig. 3), while genotype Q became the most frequent genotype, comprising 54% of the isolates (Fig. 3). Despite its circulation in the United Kingdom, genotype Q was not observed in any other European country (Fig. 2Q). Instead, mainland Europe saw the emergence of a reassortant between A(H1N1)pdm09 and the endemic H3N2 (genotype B), with the pandemic virus acquiring an NA-N2 segment (giving genotype R). This genotype was predominantly isolated in Germany, where it comprised 26% of the isolates, but was also found at a much lower prevalence in Italy and The Netherlands (Fig. 2R). Aside from these three genotypes, A(H1N1)pdm09-based viruses were found in just 3% of the samples (Fig. 1).

### Phylogenetic analyses. (i) A/swine/Scotland/410440/1994-like.

**FIG 4:**
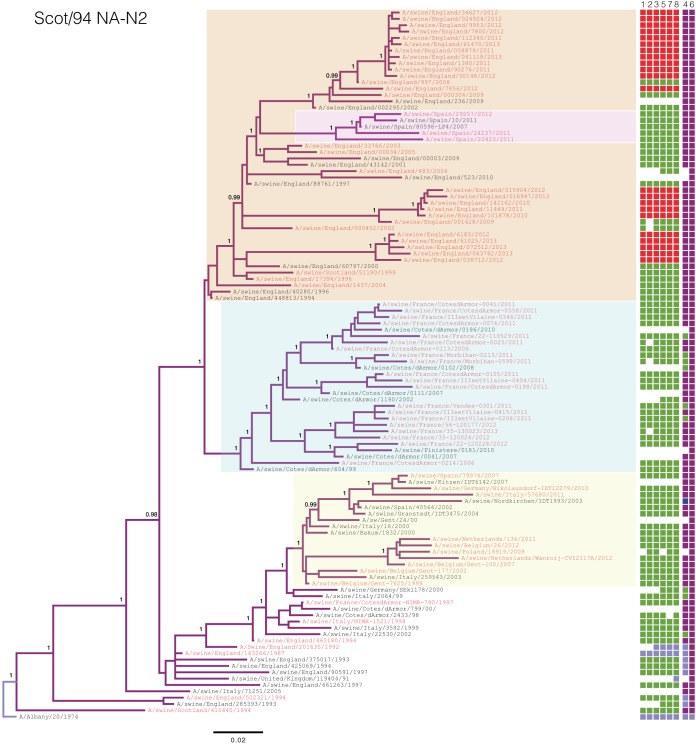
Phylogeny of the Scot/94 lineage N2 gene inferred using Bayesian analysis. Taxa sequenced by members of the ESNIP3 consortium are highlighted in red, while those in black were obtained from the Influenza Virus Resource. Colored squares to the right of each taxon name indicate the corresponding genotypes, with coloring and segment order as described for Fig. 1. White squares indicate that no sequence was available for that segment. Posterior probabilities are given at selected nodes. Colored highlights indicate well-supported circulating clades. The scale bar is given in numbers of substitutions per site.

Phylogenetic analysis of the hemagglutinin (see Fig. S1 in the supplemental material) and neuraminidase (Fig. 4) segments of the Scot/94-derived H1_hu_N2 lineage showed at least four long-lived clades of the virus circulating within Europe. Three of these were geographically structured, circulating exclusively within France (Fig. 4; blue box), Spain (Fig. 4; pink box), and the United Kingdom (Fig. 4; orange box), respectively. The fourth clade (Fig. 4; yellow box) has a different dynamic, containing viruses isolated from different countries.

Each of these four clades has further diversified into multiple subclades that are present in their geographical region. Within France, at least four subclades are observed which, on several occasions, have reassorted with EA H1_av_N1 viruses to acquire their HA-H1 segment, forming a novel reassortant genotype (genotype G). In the United Kingdom, at least three subclades are observed, each of which has reassorted with the A(H1N1)pdm09 virus to acquire their internal genes. A further reassortment event between a Spanish virus of genotype J and a United Kingdom Scot/94 virus resulted in the Spanish virus replacing its human seasonal-origin NA with Scot/94 NA to become a complete Scot/94 genotype. This then circulated in Spanish swine, apparently replacing the original virus (Fig. 4; see also Fig. S1 in the supplemental material [pink box]).

The Scot/94 hemagglutinin phylogeny shows that a distinct clade circulated in Italy. These viruses acquired an NA-N2 segment from a separate human-to-swine transmission (genotype F), with the resultant virus becoming enzootic within Italy. These viruses have evolved into at least two subclades, with one predominant between 2005 and 2010, while the other predominated after 2010.

### (ii) A/swine/Gent/1/1984-like.

**FIG 5:**
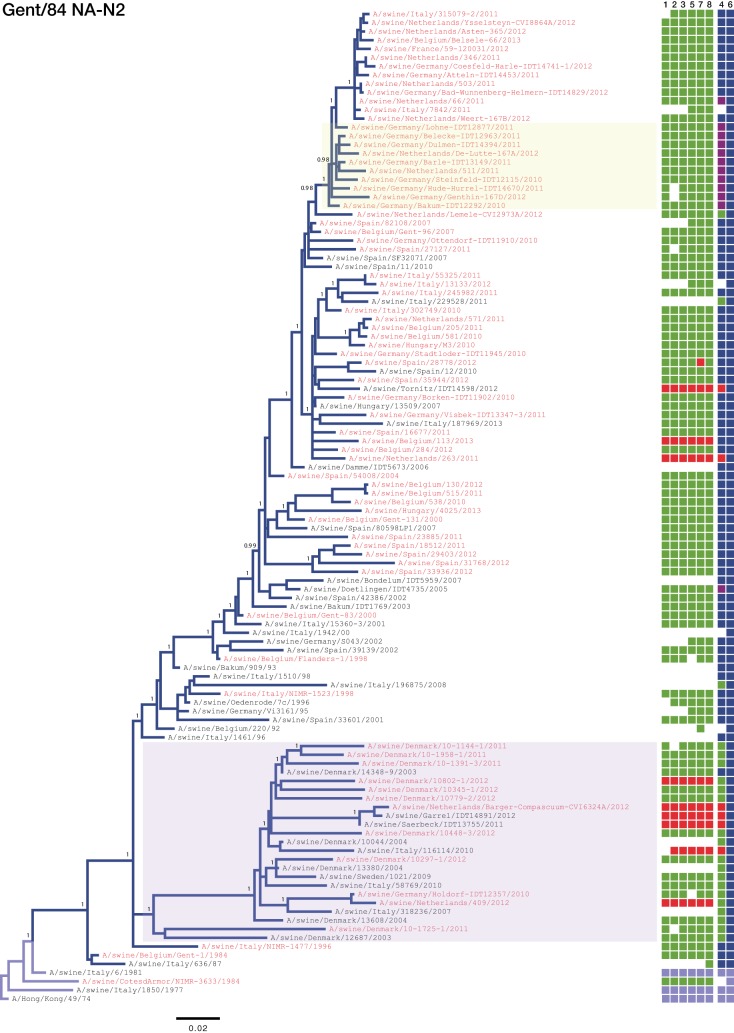
Phylogeny of the Gent/84 lineage N2 gene inferred using Bayesian analysis. Posterior probabilities are given at selected nodes, and the scale bar is given in numbers of substitutions per site. See Fig. 4 legend for other details.

Phylogenetic analysis of the neuraminidase segment for the Gent/84 H3N2 lineage shows a markedly different dynamic from that of the Scot/94 H1_hu_N2 lineage (Fig. 5). The phylogeny does not show the same maintenance of distinct lineages characteristic of the Scot/94 virus. Instead, the Gent/84 H3N2 virus (genotype B) has a more “ladder-like” phylogeny, with the higher lineage turnover and lower genetic diversity in any year characteristic of the human seasonal H3N2 virus. The virus is also geographically heterogeneous, with the short-lived lineages isolated from multiple countries concurrently.

However, a reassortment event with an EA H1_av_N1 virus resulted in the replacement of the HA-H3 segment with an HA-H1 (genotype D) (Fig. 5; purple box). This genotype became enzootic within Denmark, forming a separate clade within the Gent/84 NA-N2 phylogeny. The dynamics of the virus within this clade are more like those of the Scot/94 NA-N2, with multiple sublineages cocirculating. This genotype shows a higher propensity to reassort, with at least two sublineages emerging that have replaced their EA segments with A(H1N1)pdm09 ones (genotype R). A second A(H1N1)pdm09 reassortant that replaced its EA internal genes with pdm09 ones (genotype T) was also observed on at least two separate occasions.

### (iii) A(H1N1)pdm09.

The A(H1N1)pdm09 virus has been observed in European swine only since late 2009. As a result of this recent emergence, the genetic diversity of the virus is low, and the phylogenetic branching in the lineage is incompletely resolved, resulting in polytomies for all of the segments. To place the swine isolates in the context of the human outbreak, a molecular clock phylogeny was estimated for the internal gene cassette, combining all European swine and human isolates. The phylogeny shows the swine isolates interspersed throughout the tree, with an estimated minimum of 32 different transmissions of the virus from humans to swine (see Fig. S2 in the supplemental material). Due to the limited sampling of A(H1N1)pdm09 in swine, it is not clear from the molecular clock phylogeny whether the virus circulated enzootically in European swine or whether it was maintained through continual short-lived introductions from humans. To assess this issue statistically, Bayesian phylogenies containing human and swine isolates were inferred for the A(H1N1)pdm09 PB2 segment (see Fig. S3). Statistical testing of the association between the host species and the phylogenetic relationship ancestry using BaTS showed that the swine isolates clustered more often than expected by chance, suggesting that some of the introduced A(H1N1)pdm09 virus had been circulating within swine in Europe (Table 1). The BaTS test was repeated for the HA-H1 (see Fig. S4) and NA-N1 (see Fig. S5) segments, with the results again showing significant clustering of the swine isolates (Table 1). Together, these results suggest that both the internal genes and the external genes of the A(H1N1)pdm09 virus are clustering more than expected by chance.

**TABLE 1:** Statistical support for the association of host species with ancestry for the HA-H1, NA-N1, and PB2 segments of the A(H1N1)pdm09 lineage^*a*^

Segment	Statistic	Observed value			Null value			Significance (*P*)
		Mean	Lower 95% CI	Upper 95% CI	Mean	Lower 95% CI	Upper 95% CI	
HA-H1	AI	6.369	5.287	7.432	9.022	8.019	10.084	0.039
	PS	35.842	34	37	40.909	39.477	41.750	<0.01
NA-N1	AI	4.605	3.561	5.714	8.134	7.264	9.017	<0.01
	PS	32.886	31	35	45.541	44.012	46.573	<0.01
PB2	AI	4.883	3.987	5.804	6.925	5.951	7.792	<0.01
	PS	28.680	28	30	38.070	36.591	38.858	<0.01

a AI, association index; PS, parsimony score; CI, Bayesian credible interval.

### Reassortant genotype of public-health interest.

A genotype of particular note is the triple reassortant isolated in Spain in 2012 containing Gent/84 external glycoproteins and EA internal genes but an A(H1N1)pdm09 matrix segment (A/swine/Spain/28778/2012). This genetic makeup is comparable to that of the North American A(H3N2)v strain that has been associated with multiple swine-to-human zoonoses in North American swine fairs (44); both contain human-derived H3 and N2 glycoproteins that have since evolved within swine, and both contain internal gene cassettes with an acquired pdm09 matrix protein. This constellation therefore poses a potential public health risk, particularly as its external glycoproteins have been antigenically evolving within swine for over 30 years, so humans will likely be immunologically naive against the virus. Because of the possible public health interest in this genotype, this isolate was reextracted and resequenced, confirming its makeup.

## DISCUSSION

The genomic characterization of 290 European swIAV, of which 243 were sequenced as part of this study, is reported here. These genomes define the diversity of swIAV across Europe between 2009 and 2013, during which time the A(H1N1)pdm09 lineage was introduced back into swine through reverse zoonosis. The introduction of this new swIAV lineage into European swine increased the complexity of the circulating genotypes, resulting in an increase in the number of reassortment possibilities. In total, 23 different genotypes were observed among 278 genomes from European swine. In contrast, a study by Liang et al. in southern China over a comparable time period found 29 genotypes among 387 genomes (21). This reduced reassortment diversity in European swIAV is a result of both a smaller number of genotypes and a bias toward fewer genotypes. Furthermore, reassortment involving the IGC was rare in European swIAV, with just three (1%) isolates found to contain a reassortant IGC. In contrast, reassortment of the internal segments was observed frequently in southern China, with multiple reassortant genotypes persisting in the swine population. This is likely to be due to the presence of the TR lineage in China, in addition to the EA and pdm09 lineages, because the majority of IGC reassortment events observed by Liang et al. involved the TR lineage (21). Only one EA/pdm09 IGC reassortant—an EA genotype with an A(H1N1)pdm09 matrix gene—was isolated recurrently in China which was also isolated in this study (genotype M). This reassortment difference extends to North America, where the TR lineage first arose and is the predominant lineage; although the frequency of interlineage reassortment within the internal gene segments was lower than for southern China, it was still considerably higher than in Europe (24).

Despite 23 distinct genotypes being found in European swine, only four (A, B, C, and P) were found to be circulating across the whole of Europe. A further six (D, E, F, G, Q, and R) were found in geographically constrained areas; i.e., they were highly frequent in a single country, with occasional outbreaks in other countries. The remaining 13 genotypes were isolated sporadically and infrequently, suggesting that these were perhaps less-fit reassortants that were identified due only to the extent of surveillance. It is likely that regular whole-genome-sequencing-based surveillance of human, swine, and avian influenza will provide a more compelling catalogue of the diversity and fitness landscape of IAV reassortants than is possible through *in vitro* studies.

The four genotypes circulating throughout Europe include the three lineages that have been enzootic and prevalent in European swine for at least 19 years: EA H1_av_N1, Gent/84 H3N2, and Scot/94 H1_hu_N2. However, these genotypes have different frequencies and dynamics across mainland Europe. While H1_av_N1 was found at a high frequency in all countries, the prevalences of H3N2 and H1_hu_N2 were inversely related. These observations are consistent with the data obtained through preliminary subtyping of the ESNIP3 samples (25). Differences between the lineages were also observed in phylogenetic analysis of the two lineages' glycoprotein segments; the H1_hu_N2 phylogeny showed greater genetic diversity at any point in time through the presence of long-lived lineages that circulate independently in different countries (Fig. 4). Conversely, the H3N2 lineage had less genetic diversity at any point in time due to its rapid turnover of short-lived lineages that were geographically diffuse (Fig. 5). This dynamic relationship between the two lineages is similar to the relationship between the A/H1N1 and A/H3N2 subtypes in humans (45) or between the B/Yamagata and B/Victoria lineages in humans (46).

The differing phylogenies of the H1_hu_N2 and H3N2 lineages give an insight into their epidemiological dynamics. The ladder-like phylogeny of H3N2 suggests that the lineage is under strong selective pressure, against which the virus fixes advantageous mutations rapidly along the trunk of the tree, with loss of the side branches that do not contain the variation (47). The cocirculation of multiple subclades of the H1_hu_N2 lineage within each country, however, suggests that the virus is not subject to the same intense selective pressures as the H3N2 lineage. This could be due to reduced cross-reactive immunity in swine between the H1_hu_N2 subclades. This difference in selective pressure on the two lineages may explain why Gent/84 H3 was found only in conjunction with Gent/84 N2, whereas Scot/94 H1 was apparently more able to reassort with the NA of other lineages (Fig. 1). Furthermore, this inverse relationship between H3N2 and H1_hu_N2 may be influenced by evolutionary dynamics and selection—in swine populations in countries such as France and the United Kingdom, the emergence of H1_hu_N2 correlated with the disappearance of H3N2. Possibly immunity to N2 had an influence, favoring selection of the lineage with greater diversity and an opportunity for selection of fitter viruses in swine. The cause of the apparent absence of such pressures in other major swine-producing countries (Belgium, Germany, The Netherlands, and Spain) where H3N2 and H1_hu_N2 coexist is unclear, though it may be due to differences in their swine production systems compared to those of the United Kingdom and France. The latter have a relatively low pig density (<90 heads/hectare of agricultural area in 2010) compared to the former (>90 heads/hectare). The Netherlands, Belgium, Spain, and Germany have among the highest swine densities in Europe, with The Netherlands having had as many as 704 heads/hectare in 2010 (http://www.fao.org/docrep/017/i3138e/i3138e07.pdf). A higher swine density has been previously shown to increase the risk of seroprevalence for influenza and as such may be associated with the cocirculation of the two genotypes (48). However, a more formal statistical assessment of the predictors is needed and will require examination of different industry structures, production systems, and vaccination usage to better understand the underlying factors influencing virus evolution.

The A(H1N1)pdm09 virus that emerged in humans in early 2009 was the third-most-frequent genotype found in swine across Europe. The first confirmed disease outbreak in European pigs was in September 2009, but it is highly possible that the virus crossed to pigs from humans earlier, after its emergence in Europe in April (43). In this study, we have estimated that at least 32 separate introductions of the A(H1N1)pdm09 virus from humans into swine have occurred in the period though 2013, and we find phylogenetic evidence that the virus circulates endemically among swine. This finding is in contrast to those of the study conducted in southern China, where the A(H1N1)pdm09 virus did not persist after each introduction and its internal genes were maintained in swine only through reassortment with the HA and NA genes of other enzootic lineages (21). However, we also observed replacement of the A(H1N1)pdm09 external glycoproteins through reassortment with enzootic lineages, notably acquiring the H1 and N2 from the H1_hu_N2 lineage (genotype C) in the United Kingdom or acquiring the N2 from the H3N2 lineage (genotype B) in Germany. The prevalence of these A(H1N1)pdm09 reassortants has previously been noted (49–52) and suggests that, as in southern China, the internal genes are highly compatible with glycoprotein segments from enzootic lineages and that the circulation of the A(H1N1)pdm09 in European swine therefore increases the reassortment potential for European swIAV.

Rates of infections in swine of the A(H1N1)pdm09 virus differed across Europe; mainland Europe was found to have an average frequency of 8%, which is in agreement with the 9% proportion found from the preliminary subtyping of European swine (25). Swine in the United Kingdom, however, have shown a near-complete replacement of their enzootic H1_av_N1 and H1_hu_N2 viruses with A(H1N1)pdm09 and pdm09-H1_hu_N2 viruses. Phylogenetic analysis of the Scot/94 H1 and N2 segments showed that at least four separate sublineages of EA-H1_hu_N2 each replaced their EA IGC with the pdm09 one, consistent with the idea that the pdm09 IGC is fitter in swine. The relative proportions of the H1_av_N1 and H1_hu_N2 subtypes have remained the same since the replacement of the EA lineage by the pdm09 one; a serological study of United Kingdom swine between 2008 and 2009 showed that H1_hu_N2 was the predominant subtype, detected in 45% of all farms, with the H1_av_N1 subtype found in approximately 21% of farms (53). Consistent with this, we showed here that, since the introduction of the pdm09 virus in United Kingdom swine, the frequency of the pdm09-H1_hu_N2 is approximately 54% whereas that of the pdm09 virus is 27%. The reasons for the difference in prevalence between mainland Europe and the United Kingdom are unclear and warrant further investigation.

A triple reassortant genotype containing an EA IGC with a pdm09 matrix gene and Gent/84 H3 and N2 segments (genotype N) was observed in a single Spanish pig (A/swine/Spain/28778/2012). Acquisition of the pdm09 matrix protein by enzootic swIAV has been previously noted in China (21) and also in the United States, where the resultant A(H3N2)v genotypes were able to infect humans (54, 55). Genotype N has a genetic makeup similar to that of A(H3N2)v, and, importantly, its external glycoproteins are derived from the Gent/84 H3N2 lineage that has been evolving in swine since the early-to-middle 1970s (1). As such, humans are likely to be immunologically naive against this virus, and it thus poses a potential public health risk. Given the level of reassortment observed in European swine, further surveillance efforts should be sought to track the emergence and potential spread of such genotypes with human-pandemic potential. Whole-genome sequencing of swIAV isolates is an important aspect of this surveillance effort, without which the dynamics of the circulating lineages cannot be determined. Furthermore, it is only through whole-genome sequencing that the rare, but potentially important, reassortants involving the IGC can be observed. However, the limited IGC reassortment indicates that preliminary subtyping of the HA and NA segments is still suitable for routine surveillance of European swIAV.

This report reveals that the emergences and drivers of virus evolution in pigs differ at the global level. The factors favoring virus emergence and selection are complex, but we show that establishment of new genotypes and lineages is complex and less frequent at the population level. Whole-system analyses performed at the virus host level, together with analysis of the influence of natural or vaccine-derived immunity, require further investigation.

## Supplementary Material

Supplemental material
