# Supplementary material for: Molecular Epidemiology and Evolution of Influenza Viruses Circulating within European Swine between 2009 and 2013
Source: J Virol. 2015 Jul 22;89(19):9920–31. doi: 10.1128/JVI.00840-15 (PMC4577897; doi:10.1128/JVI.00840-15)
Supplement: Supplemental material [file supp_89_19_9920__index.html]

Supplemental material 

# Molecular Epidemiology and Evolution of Influenza Viruses Circulating within European Swine between 2009 and 2013

## Supplemental material

- Supplemental file 1 -

  Fig. S1 (Bayesian-inferred phylogeny of the Scot/94 lineage H1 gene.)

  Fig. S2 (Bayesian-inferred molecular clock phylogeny of the concatenated internal gene cassette of the A(H1N1)pdm09 lineage.)

  Fig. S3 (Bayesian-inferred phylogeny of the A(H1N1)pdm09 lineage PB2 gene.)

  Fig. S4 (Bayesian-inferred phylogeny of the A(H1N1)pdm09 lineage H1 gene.)

  Fig. S5 (Bayesian-inferred phylogeny of the A(H1N1)pdm09 lineage N1 gene.)

  Table S1 (Overview of genome completion for the isolates used in this study.)

  PDF, 489K
