# Supplementary material for: Molecular Epidemiology and Evolution of Influenza Viruses Circulating within European Swine between 2009 and 2013
Source: J Virol. 2015 Jul 22;89(19):9920–31. doi: 10.1128/JVI.00840-15 (PMC4577897; doi:10.1128/JVI.00840-15)
Supplement: Supplemental material [file JVI.00840-15_zjv999090819so1.pdf]

Supplementary Figure 1: Scot/94 HA-H1

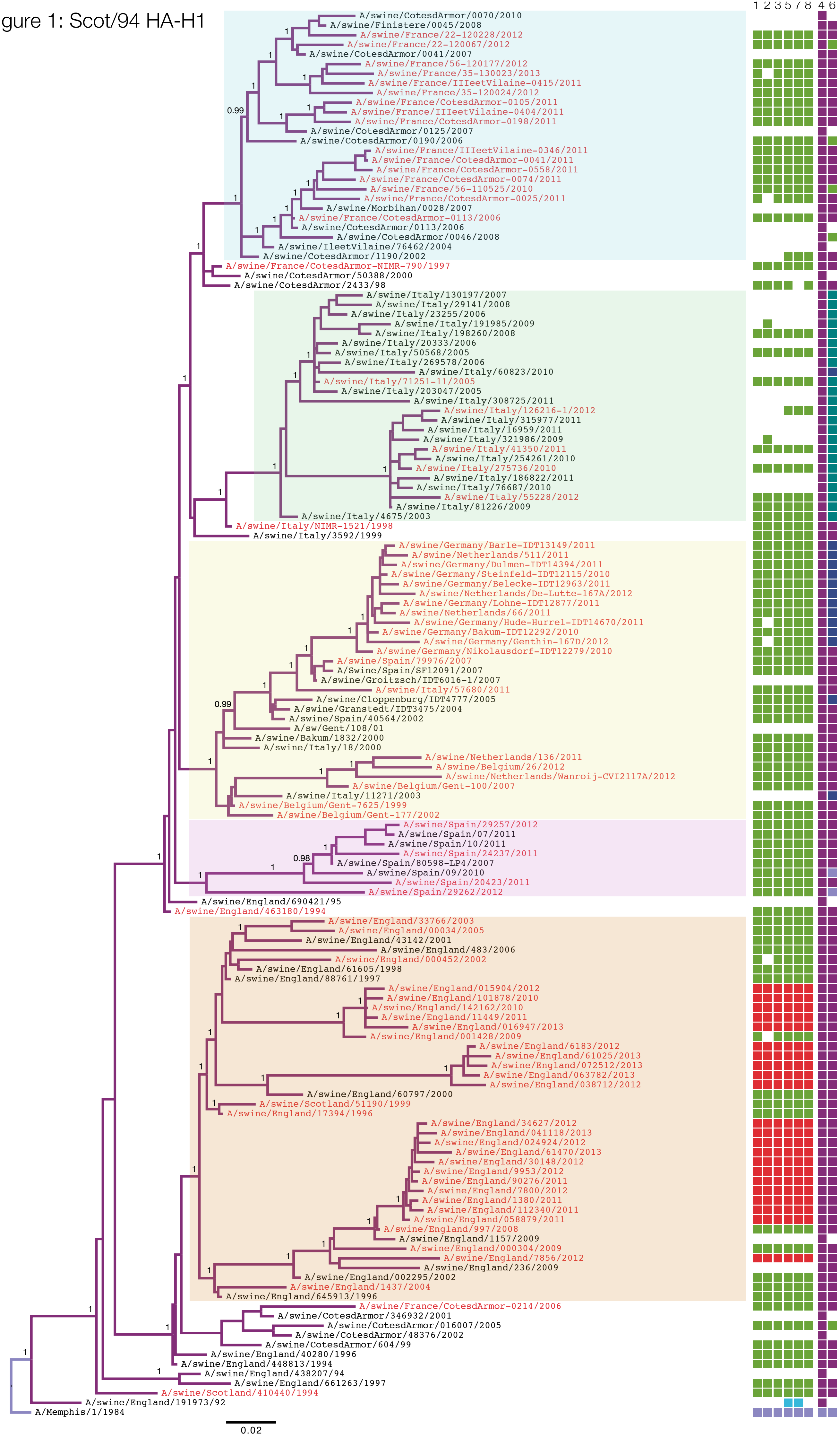

Supplementary Figure 1. Bayesian-inferred phylogeny of the Scot/94 lineage H1 gene. Taxa sequenced through the ESNIP3 consortium are highlighted in red, while those in black were obtained from the Influenza Virus Resource. Colored squares to the right of each taxa indicate its genotype, with coloring and segment order as in Figure 1. White squares indicate that no sequence was available for that segment. Posterior probabilities are given at selected nodes. Colored highlights indicate well-supported circulating clades. The scale bar is given in substitutions per site.

Supplementary Figure 2: A(H1N1)pdm09 IGC

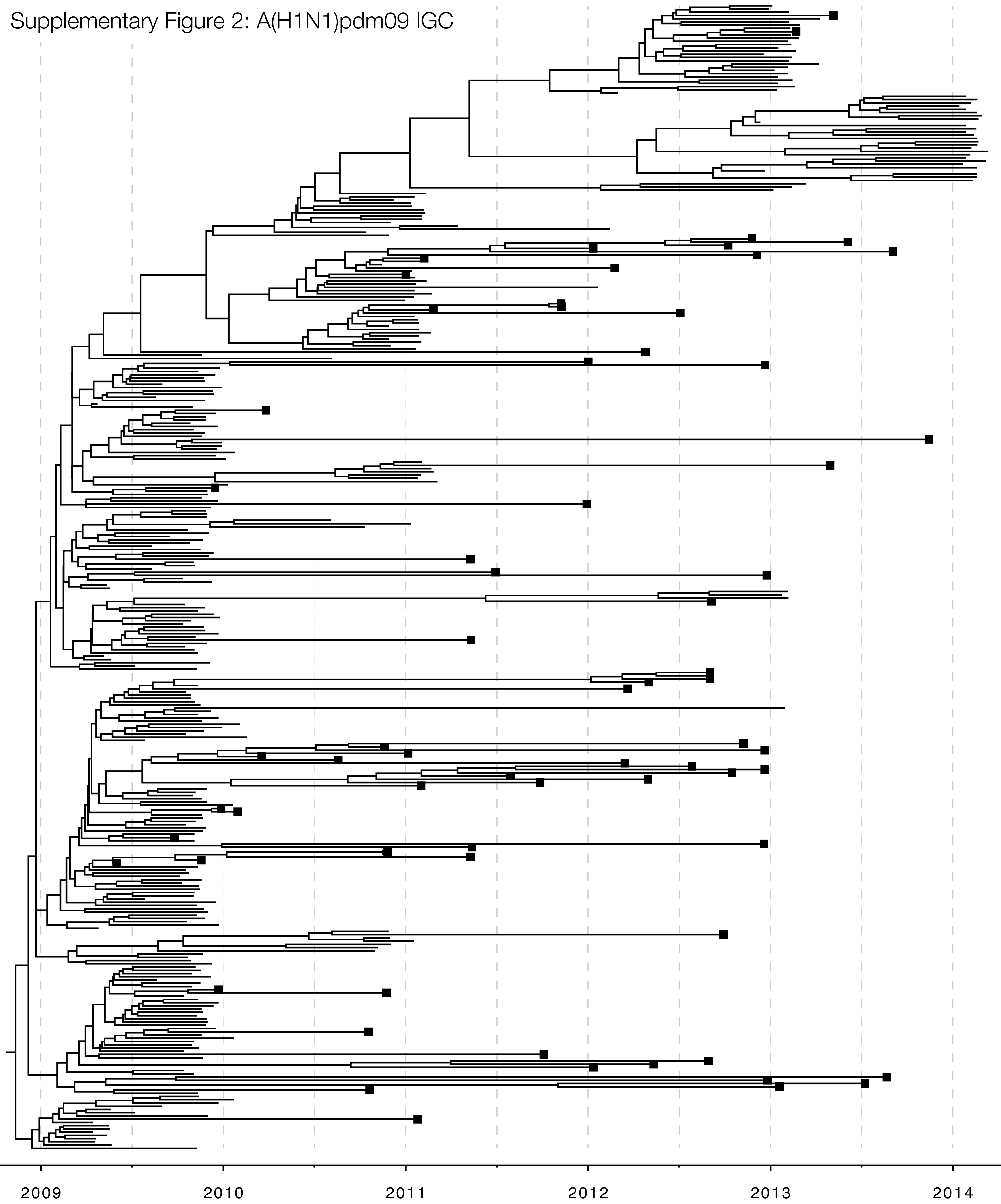

Supplementary Figure 2. Bayesian-inferred molecular clock phylogeny of the concatenated internal gene cassette of the A(H1N1)pdm09 lineage. Black squares at the tips indicate isolates derived from swine, while those without were taken from humans.

Supplementary Figure 3:
A(H1N1)pdm09 PB2

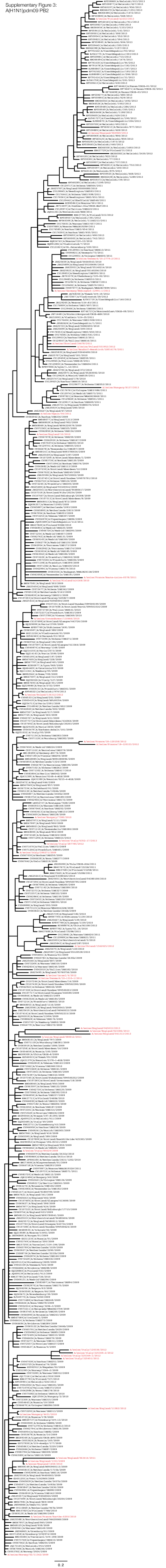

Supplementary Figure 3. Bayesian-inferred phylogeny of the A(H1N1)pdm09 lineage PB2 gene. Isolate names in red indicate isolates derived from a swine host, whilst those in black were taken from a human host. The scale bar is given in substitutions per site.

Supplementary Figure 4
A(H1N1)pdm09 HA

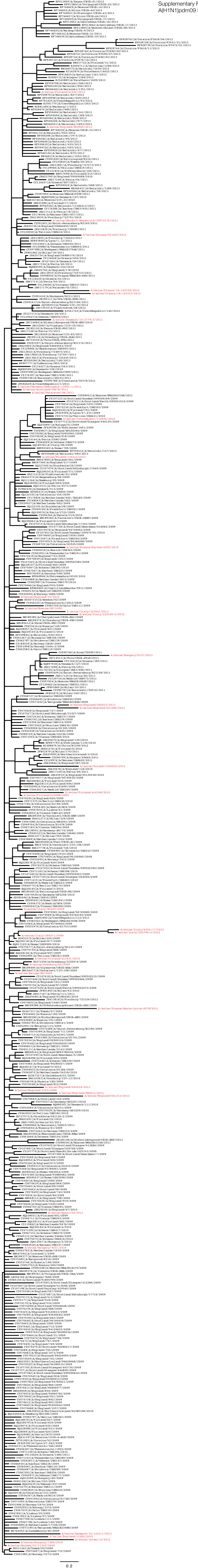

Supplementary Figure 4. Bayesian-inferred phylogeny of the A(H1N1)pdm09 lineage H1 gene. Isolate names in red indicate isolates derived from a swine host, whilst those in black were taken from a human host. The scale bar is given in substitutions per site.

Supplementary Figure 5:
A(H1N1)pdm09 NA

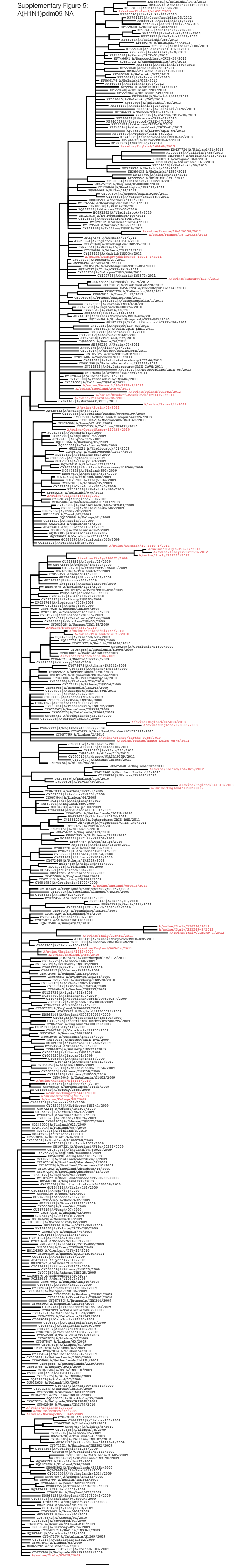

Supplementary Figure 5. Bayesian-inferred phylogeny of the A(H1N1)pdm09 lineage N1 gene. Isolate names in red indicate isolates derived from a swine host, whilst those in black were taken from a human host. The scale bar is given in substitutions per site.

Strain	Country	Year	PB2	PB1	PA	NP	MP	NS	HA	NA	Genotype
A/swine/Belgium/553/2010	Belgium	2010	EA	EA	EA	EA	EA	EA	EA	EA	A
A/swine/Belgium/560/2010	Belgium	2010	EA	EA	EA	EA	EA	EA	EA	EA	A
A/swine/Belgium/455/2011	Belgium	2011	EA	EA	EA	EA	EA	EA	EA	EA	A
A/swine/Belgium/387/2011	Belgium	2011	EA	EA	EA	EA	EA	EA	EA	EA	A
A/swine/Belgium/231/2011	Belgium	2011	EA	EA	EA	EA	EA	EA	EA	EA	A
A/swine/Belgium/80/2011	Belgium	2011	EA	EA	EA	EA	EA	EA	EA	EA	A
A/swine/Belgium/628/2010	Belgium	2010	EA	EA	EA	EA	EA	EA	EA	EA	A
A/swine/Belgium/37/2011	Belgium	2011	EA	EA	EA	EA	EA	EA	EA	EA	A
A/swine/Belgium/Deinze-393/2012	Belgium	2012	EA	EA	EA	EA	EA	EA	EA	EA	A
A/swine/Belgium/Minderhout-348/2012	Belgium	2012	EA	EA	EA	EA	EA	EA	EA	EA	A
A/swine/Belgium/Glabbeek-287/2012	Belgium	2012	EA	EA	EA	EA	EA	EA	EA	EA	A
A/swine/Belgium/Heist-op-den-Berg-363/2012	Belgium	2012	EA	EA	EA	EA	EA	EA	EA	EA	A
A/swine/Belgium/Lichtervelde-57/2013	Belgium	2013	EA	EA	EA	EA	EA	EA	EA	EA	A
A/swine/Belgium/111/2013	Belgium	2013	EA	EA	EA	EA	EA	EA	EA	EA	A
A/swine/Denmark/10-1048-2/2011	Denmark	2011	EA		EA	EA	EA	EA	EA	EA	A
A/swine/Denmark/10-2526-1/2011	Denmark	2011	EA	EA	EA	EA	EA	EA	EA	EA	A
A/swine/Denmark/1085-1/2012	Denmark	2012	EA	EA	EA	EA	EA	EA	EA	EA	A
A/swine/Denmark/10169-3/2012	Denmark	2012	EA	EA	EA	EA	EA	EA	EA	EA	A
A/swine/Denmark/10146-1/2012	Denmark	2012	EA	EA	EA	EA	EA	EA	EA	EA	A
A/swine/England/1251/2011	England	2011	EA	EA	EA	EA	EA	EA	EA	EA	A
A/swine/England/024079/2013	England	2013	EA	EA	EA	EA	EA	EA	EA	EA	A
A/swine/France/CotesdArmor-0275/2010	France	2010	EA	EA	EA	EA	EA	EA	EA	EA	A
A/swine/France/Finistere-0011/2011	France	2011	EA	EA	EA	EA	EA	EA	EA	EA	A
A/swine/France/IIIeetVilaine-0187/2011	France	2011	EA	EA	EA	EA	EA	EA	EA	EA	A
A/swine/France/Morbihan-0294/2011	France	2011	EA	EA	EA	EA	EA	EA	EA	EA	A
A/swine/France/LoireAtlantique-0405/2011	France	2011	EA	EA	EA	EA	EA	EA	EA	EA	A
A/swine/France/IIIeetVilaine-0354/2011	France	2011	EA	EA	EA	EA	EA	EA	EA	EA	A
A/swine/France/IIIeetVilaine-0266/2011	France	2011	EA	EA	EA	EA	EA	EA	EA	EA	A
A/swine/France/Morbihan-0285/2011	France	2011	EA	EA	EA	EA	EA	EA	EA	EA	A
A/swine/France/CotesdArmor-0147/2011	France	2011	EA	EA	EA	EA	EA	EA	EA	EA	A
A/swine/France/CotesdArmor-0292/2011	France	2011	EA	EA	EA	EA	EA	EA	EA	EA	A
A/swine/France/CotesdArmor-0023/2011	France	2011	EA		EA	EA	EA	EA	EA	EA	A
A/swine/France/29-120064/2012	France	2012	EA	EA	EA	EA	EA	EA	EA	EA	A
A/swine/France/CotesdArmor-0388/2009	France	2009	EA	EA	EA	EA	EA	EA	EA	EA	A
A/swine/France/IIIeetVilaine-0208/2011	France	2011	EA	EA	EA	EA	EA	EA	EA	EA	A
A/swine/France/CotesdArmor-0364/2011	France	2011	EA		EA	EA	EA	EA	EA	EA	A
A/swine/France/22-120146/2012	France	2012	EA	EA	EA	EA	EA	EA	EA	EA	A
A/swine/France/50-120350/2012	France	2012	EA	EA	EA	EA	EA	EA	EA	EA	A
A/swine/France/22-120340/2012	France	2012	EA	EA	EA	EA	EA	EA	EA	EA	A
A/swine/France/29-120326/2012	France	2012	EA	EA	EA	EA	EA	EA	EA	EA	A
A/swine/France/01-130054/2013	France	2013	EA	EA	EA	EA	EA	EA	EA	EA	A

A/swine/Germany/Hagstedt-IDT11951/2010	Germany	2010	EA	EA	EA	EA	EA	EA	EA	EA	A
A/swine/Germany/Lohne-IDT12137/2010	Germany	2010	EA	EA	EA	EA	EA	EA	EA	EA	A
A/swine/Germany/Ostbevern-IDT13211/2011	Germany	2011	EA	EA	EA	EA	EA	EA	EA	EA	A
A/swine/Germany/Wunnenberg-IDT13220/2011	Germany	2011	EA	EA	EA	EA	EA	EA	EA	EA	A
A/swine/Germany/Reinberg-IDT14457-1/2012	Germany	2012	EA	EA	EA	EA	EA	EA	EA	EA	A
A/swine/Germany/Ahlen-Dolberg-IDT14631/2012	Germany	2012	EA	EA	EA	EA	EA	EA	EA	EA	A
A/swine/Germany/Ellerbrock-IDT14696/2012	Germany	2012	EA	EA	EA	EA	EA	EA	EA	EA	A
A/swine/Italy/302593/2010	Italy	2010	EA	EA	EA	EA	EA	EA	EA	EA	A
A/swine/Italy/317775/2010	Italy	2010	EA	EA	EA	EA	EA	EA	EA	EA	A
A/swine/Italy/28762-3/2013	Italy	2013	EA	EA	EA	EA	EA	EA	EA	EA	A
A/swine/Italy/224790/2012	Italy	2012	EA			EA	EA	EA	EA	EA	A
A/swine/Italy/233405/2012	Italy	2012	EA		EA	EA	EA	EA	EA	EA	A
A/swine/Italy/7821/2011	Italy	2011	EA	EA	EA	EA	EA	EA	EA	EA	A
A/swine/Netherlands/186/2011	Netherlands	2011	EA	EA	EA	EA	EA	EA	EA	EA	A
A/swine/Netherlands/51/2012	Netherlands	2012	EA	EA	EA	EA	EA	EA	EA	EA	A
A/swine/Netherlands/37/2012	Netherlands	2012	EA	EA	EA	EA	EA	EA	EA	EA	A
A/swine/Netherlands/530/2011	Netherlands	2011	EA	EA	EA	EA	EA	EA	EA	EA	A
A/swine/Netherlands/75/2011	Netherlands	2011	EA	EA	EA	EA	EA	EA	EA	EA	A
A/swine/Netherlands/60/2012	Netherlands	2012	EA	EA	EA	EA	EA	EA	EA	EA	A
A/swine/Netherlands/174/2011	Netherlands	2011	EA	EA	EA	EA	EA	EA	EA	EA	A
A/swine/Netherlands/12/2012	Netherlands	2012	EA	EA	EA	EA	EA	EA	EA	EA	A
A/swine/Netherlands/Vlierden-220/2012	Netherlands	2012	EA	EA	EA	EA	EA	EA	EA	EA	A
A/swine/Netherlands/Hoogeloon-167C/2012	Netherlands	2012	EA	EA	EA	EA	EA	EA	EA	EA	A
A/swine/Netherlands/Kootwijkerbroek-364/2012	Netherlands	2012	EA	EA	EA	EA	EA	EA	EA	EA	A
A/swine/Netherlands/Wijhe-371/2012	Netherlands	2012	EA	EA			EA	EA	EA	EA	A
A/swine/Netherlands/Rhezerveen-CVI9121A/2012	Netherlands	2012	EA	EA	EA	EA	EA	EA	EA	EA	A
A/swine/Netherlands/Wanroij-CVI2115A/2012	Netherlands	2012	EA	EA	EA	EA	EA	EA	EA	EA	A
A/swine/Netherlands/Mill-CVI2617A/2012	Netherlands	2012	EA	EA	EA	EA	EA	EA	EA	EA	A
A/swine/Poland/00109/2010	Poland	2010	EA	EA	EA	EA	EA	EA	EA	EA	A
A/swine/Poland/16315/2011	Poland	2011	EA	EA	EA	EA	EA	EA	EA	EA	A
A/swine/Poland/1744/2012	Poland	2012	EA	EA	EA	EA	EA	EA	EA	EA	A
A/swine/Poland/321/2012	Poland	2012	EA	EA	EA	EA	EA	EA	EA	EA	A
A/swine/Poland/18275/2012	Poland	2012	EA	EA	EA	EA	EA	EA	EA	EA	A
A/swine/Poland/006801/2013	Poland	2013	EA	EA	EA	EA	EA	EA	EA	EA	A
A/swine/Scotland/034632/2012	Scotland	2012	EA	EA	EA	EA	EA	EA	EA	EA	A
A/swine/Spain/22013/2011	Spain	2011	EA	EA	EA	EA	EA	EA	EA	EA	A
A/swine/Spain/23998/2011	Spain	2011	EA	EA	EA	EA	EA	EA	EA	EA	A
A/swine/Spain/33903/2012	Spain	2012	EA	EA	EA	EA	EA	EA	EA	EA	A
A/swine/Belgium/205/2011	Belgium	2011	EA	EA	EA	EA	EA	EA	Gent84	Gent84	B
A/swine/Belgium/538/2010	Belgium	2010	EA	EA	EA	EA	EA	EA	Gent84	Gent84	B
A/swine/Belgium/581/2010	Belgium	2010	EA	EA	EA	EA	EA	EA	Gent84	Gent84	B
A/swine/Belgium/130/2012	Belgium	2012	EA	EA	EA	EA	EA	EA	Gent84	Gent84	B

A/swine/Belgium/515/2011	Belgium	2011	EA	EA	EA	EA	EA	EA	Gent84	Gent84	B
A/swine/Belgium/284/2012	Belgium	2012	EA	EA	EA	EA	EA	EA	Gent84	Gent84	B
A/swine/Belgium/Belsele-66/2013	Belgium	2013	EA	EA	EA	EA	EA	EA	Gent84	Gent84	B
A/swine/France/59-120031/2012	France	2012	EA	EA	EA	EA	EA	EA	Gent84	Gent84	B
A/swine/Germany/Borken-IDT11902/2010	Germany	2010	EA	EA	EA	EA	EA	EA	Gent84	Gent84	B
A/swine/Germany/Ottendorf-IDT11910/2010	Germany	2010	EA	EA	EA	EA	EA	EA	Gent84	Gent84	B
A/swine/Germany/Stadtloder-IDT11945/2010	Germany	2010	EA	EA	EA	EA	EA	EA	Gent84	Gent84	B
A/swine/Germany/Atteln-IDT14453/2011	Germany	2011	EA	EA	EA	EA	EA	EA	Gent84	Gent84	B
A/swine/Germany/Bad-Wunnenberg-Helmern-IDT14829/2012	Germany	2012	EA	EA	EA	EA	EA	EA	Gent84	Gent84	B
A/swine/Germany/Visbek-IDT13347-3/2011	Germany	2011	EA	EA	EA	EA	EA	EA	Gent84	Gent84	B
A/swine/Germany/Coesfeld-Harle-IDT14741-1/2012	Germany	2012	EA	EA	EA	EA	EA	EA	Gent84	Gent84	B
A/swine/Hungary/M3/2010	Hungary	2010	EA	EA	EA	EA	EA	EA	Gent84	Gent84	B
A/swine/Hungary/4025/2013	Hungary	2013	EA	EA	EA	EA	EA	EA	Gent84	Gent84	B
A/swine/Italy/302749/2010	Italy	2010	EA	EA	EA	EA	EA	EA	Gent84	Gent84	B
A/swine/Italy/55325/2011	Italy	2011	EA	EA	EA	EA	EA	EA	Gent84	Gent84	B
A/swine/Italy/315079-2/2011	Italy	2011		EA	EA	EA	EA	EA	Gent84	Gent84	B
A/swine/Italy/245982/2011	Italy	2011	EA	EA	EA	EA	EA	EA	Gent84	Gent84	B
A/swine/Netherlands/571/2011	Netherlands	2011	EA	EA	EA	EA	EA	EA	Gent84	Gent84	B
A/swine/Netherlands/346/2011	Netherlands	2011	EA	EA	EA	EA	EA	EA	Gent84	Gent84	B
A/swine/Netherlands/503/2011	Netherlands	2011	EA	EA	EA	EA	EA	EA	Gent84	Gent84	B
A/swine/Netherlands/Weert-167B/2012	Netherlands	2012	EA	EA	EA	EA	EA	EA	Gent84	Gent84	B
A/swine/Netherlands/Asten-365/2012	Netherlands	2012	EA	EA	EA	EA	EA	EA	Gent84	Gent84	B
A/swine/Netherlands/Ysselsteyn-CVI8864A/2012	Netherlands	2012	EA	EA	EA	EA	EA	EA	Gent84	Gent84	B
A/swine/Spain/16677/2011	Spain	2011	EA	EA	EA	EA	EA	EA	Gent84	Gent84	B
A/swine/Spain/18512/2011	Spain	2011	EA	EA	EA	EA	EA	EA	Gent84	Gent84	B
A/swine/Spain/23885/2011	Spain	2011	EA	EA	EA	EA	EA	EA	Gent84	Gent84	B
A/swine/Spain/27127/2011	Spain	2011	EA		EA	EA	EA	EA	Gent84	Gent84	B
A/swine/Spain/29403/2012	Spain	2012	EA	EA	EA	EA	EA	EA	Gent84	Gent84	B
A/swine/Spain/33936/2012	Spain	2012	EA	EA	EA	EA	EA	EA	Gent84	Gent84	B
A/swine/Spain/35944/2012	Spain	2012	EA	EA	EA	EA	EA	EA	Gent84	Gent84	B
A/swine/Spain/31768/2012	Spain	2012	EA	EA	EA	EA	EA	EA	Gent84	Gent84	B
A/swine/Belgium/26/2012	Belgium	2012	EA	EA	EA	EA	EA	EA	Scot94	Scot94	C
A/swine/England/000304/2009	England	2009	EA	EA	EA	EA	EA	EA	Scot94	Scot94	C
A/swine/England/001428/2009	England	2009	EA		EA	EA	EA	EA	Scot94	Scot94	C
A/swine/France/IIIeetVilaine-0415/2011	France	2011	EA	EA	EA	EA	EA	EA	Scot94	Scot94	C
A/swine/France/CotesdArmor-0198/2011	France	2011	EA	EA	EA	EA	EA	EA	Scot94	Scot94	C
A/swine/France/IIIeetVilaine-0346/2011	France	2011	EA	EA	EA	EA	EA	EA	Scot94	Scot94	C
A/swine/France/CotesdArmor-0025/2011	France	2011	EA		EA	EA	EA	EA	Scot94	Scot94	C
A/swine/France/CotesdArmor-0041/2011	France	2011	EA	EA	EA	EA	EA	EA	Scot94	Scot94	C
A/swine/France/CotesdArmor-0105/2011	France	2011	EA	EA	EA	EA	EA	EA	Scot94	Scot94	C
A/swine/France/CotesdArmor-0074/2011	France	2011	EA	EA	EA	EA	EA	EA	Scot94	Scot94	C
A/swine/France/IIIeetVilaine-0404/2011	France	2011	EA	EA	EA	EA	EA	EA	Scot94	Scot94	C

A/swine/France/35-120024/2012	France	2012	EA	EA	EA	EA	EA	EA	Scot94	Scot94	C
A/swine/France/CotesdArmor-0558/2011	France	2011	EA	EA	EA	EA	EA	EA	Scot94	Scot94	C
A/swine/France/22-120228/2012	France	2012	EA	EA	EA	EA	EA	EA	Scot94	Scot94	C
A/swine/France/56-120177/2012	France	2012	EA	EA	EA	EA	EA	EA	Scot94	Scot94	C
A/swine/France/35-130023/2013	France	2013	EA		EA	EA	EA	EA	Scot94	Scot94	C
A/swine/Germany/Nikolausdorf-IDT12279/2010	Germany	2010	EA	EA	EA	EA	EA	EA	Scot94	Scot94	C
A/swine/Italy/57680/2011	Italy	2011	EA	EA	EA	EA	EA	EA	Scot94	Scot94	C
A/swine/Netherlands/136/2011	Netherlands	2011	EA	EA	EA	EA	EA	EA	Scot94	Scot94	C
A/swine/Netherlands/Wanroij-CVI2117A/2012	Netherlands	2012	EA	EA	EA	EA	EA	EA	Scot94	Scot94	C
A/swine/Spain/20423/2011	Spain	2011	EA	EA	EA	EA	EA	EA	Scot94	Scot94	C
A/swine/Spain/24237/2011	Spain	2011	EA	EA	EA	EA	EA	EA	Scot94	Scot94	C
A/swine/Spain/29257/2012	Spain	2012	EA	EA	EA	EA	EA	EA	Scot94	Scot94	C
A/swine/Denmark/10-1144-1/2011	Denmark	2011	EA		EA	EA	EA	EA	EA	Gent84	D
A/swine/Denmark/10-1725-1/2011	Denmark	2011	EA		EA	EA	EA	EA	EA	Gent84	D
A/swine/Denmark/10-1958-1/2011	Denmark	2011	EA	EA	EA	EA	EA	EA	EA	Gent84	D
A/swine/Denmark/10297-1/2012	Denmark	2012	EA	EA	EA	EA	EA	EA	EA	Gent84	D
A/swine/Denmark/10448-3/2012	Denmark	2012	EA	EA	EA	EA	EA	EA	EA	Gent84	D
A/swine/Denmark/10779-2/2012	Denmark	2012	EA	EA	EA	EA	EA	EA	EA	Gent84	D
A/swine/Denmark/10345-1/2012	Denmark	2012	EA	EA	EA	EA	EA	EA	EA	Gent84	D
A/swine/Denmark/10-1391-3/2011	Denmark	2011	EA	EA	EA	EA	EA	EA	EA	Gent84	D
A/swine/Germany/Holdorf-IDT12357/2010	Germany	2010	EA	EA	EA		EA	EA	EA	Gent84	D
A/swine/Netherlands/Lemele-CVI2973A/2012	Netherlands	2012	EA	EA	EA	EA	EA	EA	EA	Gent84	D
A/swine/Germany/Steinfeld-IDT12115/2010	Germany	2010	EA	EA	EA	EA	EA	EA	Scot94	Gent84	E
A/swine/Germany/Bakum-IDT12292/2010	Germany	2010	EA	EA	EA	EA	EA	EA	Scot94	Gent84	E
A/swine/Germany/Lohne-IDT12877/2011	Germany	2011	EA	EA	EA	EA	EA	EA	Scot94	Gent84	E
A/swine/Germany/Belecke-IDT12963/2011	Germany	2011	EA	EA	EA	EA	EA	EA	Scot94	Gent84	E
A/swine/Germany/Barle-IDT13149/2011	Germany	2011	EA	EA	EA	EA	EA	EA	Scot94	Gent84	E
A/swine/Germany/Dulmen-IDT14394/2011	Germany	2011	EA	EA	EA	EA	EA	EA	Scot94	Gent84	E
A/swine/Germany/Hude-Hurrel-IDT14670/2011	Germany	2011	EA		EA	EA	EA	EA	Scot94	Gent84	E
A/swine/Germany/Genthin-167D/2012	Germany	2012	EA		EA	EA	EA	EA	Scot94	Gent84	E
A/swine/Netherlands/De-Lutte-167A/2012	Netherlands	2012	EA	EA	EA	EA	EA	EA	Scot94	Gent84	E
A/swine/Netherlands/66/2011	Netherlands	2011	EA	EA	EA	EA	EA	EA	Scot94	Gent84	E
A/swine/Netherlands/511/2011	Netherlands	2011	EA	EA	EA	EA	EA	EA	Scot94	Gent84	E
A/swine/Italy/41350/2011	Italy	2011	EA	EA	EA	EA	EA	EA	Scot94	It-N2	F
A/swine/Italy/275736/2010	Italy	2010	EA	EA	EA	EA	EA	EA	Scot94	It-N2	F
A/swine/Italy/55228/2012	Italy	2012	EA	EA	EA	EA	EA	EA	Scot94	It-N2	F
A/swine/Italy/126216-1/2012	Italy	2012				EA	EA	EA	Scot94	It-N2	F
A/swine/France/Morbihan-0213/2011	France	2011	EA	EA	EA	EA	EA	EA	EA	Scot94	G
A/swine/France/Vendee-0301/2011	France	2011	EA	EA	EA	EA	EA	EA	EA	Scot94	G
A/swine/France/Morbihan-0599/2011	France	2011	EA		EA	EA	EA	EA	EA	Scot94	G
A/swine/France/22-110529/2011	France	2011	EA	EA	EA	EA	EA	EA	EA	Scot94	G
A/swine/France/56-110525/2010	France	2010	EA	EA	EA	EA	EA	EA	Scot94	EA	H

A/swine/France/22-120067/2012	France	2012	EA	EA	EA	EA	EA	EA	Scot94	EA	H
A/swine/Denmark/10302-2/2012	Denmark	2012	EA		EA	EA	EA	EA	EA	huH3N2	I
A/swine/France/37-120345/2012	France	2012	EA	EA	EA	EA	EA	EA	EA	huH3N2	I
A/swine/Spain/29262/2012	Spain	2012	EA	EA	EA	EA	EA	EA	Scot94	huH3N2	J
A/swine/Italy/16235/2013	Italy	2013	EA		EA	EA	EA	EA	EA	It-N2	L
A/swine/Italy/303612/2011	Italy	2011	EA	EA	EA	EA	pdm09	EA	EA	EA	M
A/swine/Spain/28778/2012	Spain	2012	EA	EA	EA	EA	pdm09	EA	Gent84	Gent84	N
A/swine/Denmark/10-2779-2/2011	Denmark	2011	pdm09	pdm09	pdm09	pdm09	pdm09	pdm09	pdm09	pdm09	P
A/swine/Denmark/10-1310-1/2011	Denmark	2011	pdm09	pdm09	pdm09	pdm09	pdm09	pdm09	pdm09	pdm09	P
A/swine/England/063414/2011	England	2011	pdm09	pdm09	pdm09	pdm09	pdm09	pdm09	pdm09	pdm09	P
A/swine/England/080012/2011	England	2011	pdm09	pdm09	pdm09	pdm09	pdm09	pdm09	pdm09	pdm09	P
A/swine/England/045033/2013	England	2013	pdm09	pdm09	pdm09	pdm09	pdm09	pdm09	pdm09	pdm09	P
A/swine/England/041313/2013	England	2013	pdm09	pdm09	pdm09	pdm09	pdm09	pdm09	pdm09	pdm09	P
A/swine/England/11582/2012	England	2012	pdm09	pdm09	pdm09	pdm09	pdm09	pdm09	pdm09	pdm09	P
A/swine/England/060969/2013	England	2013	pdm09	pdm09	pdm09	pdm09	pdm09	pdm09	pdm09	pdm09	P
A/swine/England/023386/2013	England	2013	pdm09	pdm09	pdm09	pdm09	pdm09	pdm09	pdm09	pdm09	P
A/swine/England/1353/2009	England	2009	pdm09	pdm09	pdm09	pdm09	pdm09	pdm09	pdm09	pdm09	P
A/swine/Finland/si4168/2010	Finland	2010	pdm09	pdm09	pdm09	pdm09	pdm09	pdm09	pdm09	pdm09	P
A/swine/Finland/si632/2013	Finland	2013	pdm09	pdm09	pdm09	pdm09	pdm09	pdm09	pdm09	pdm09	P
A/swine/France/Sarthe-0255/2010	France	2010	pdm09	pdm09	pdm09	pdm09	pdm09	pdm09	pdm09	pdm09	P
A/swine/France/Haute-Loire-0578/2011	France	2011	pdm09	pdm09	pdm09	pdm09	pdm09	pdm09	pdm09	pdm09	P
A/swine/France/18-120158/2012	France	2012	pdm09	pdm09	pdm09	pdm09	pdm09	pdm09	pdm09	pdm09	P
A/swine/Germany/Ebbinghof-12991-1/2011	Germany	2011	pdm09	pdm09	pdm09	pdm09	pdm09	pdm09	pdm09	pdm09	P
A/swine/Hungary/4431/2010	Hungary	2010	pdm09	pdm09	pdm09	pdm09	pdm09	pdm09	pdm09	pdm09	P
A/swine/Hungary/S137/2013	Hungary	2013	pdm09	pdm09	pdm09	pdm09	pdm09	pdm09	pdm09	pdm09	P
A/swine/Israel/4/2012	Israel	2012	pdm09	pdm09	pdm09	pdm09	pdm09	pdm09	pdm09	pdm09	P
A/swine/Italy/6352-17/2013	Italy	2013	pdm09	pdm09	pdm09	pdm09	pdm09	pdm09	pdm09	pdm09	P
A/swine/Poland/1562025/2012	Poland	2012	pdm09	pdm09	pdm09	pdm09	pdm09	pdm09	pdm09	pdm09	P
A/swine/Poland/134312/2012	Poland	2012	pdm09	pdm09	pdm09	pdm09	pdm09	pdm09	pdm09	pdm09	P
A/swine/Poland/031952/2012	Poland	2012	pdm09	pdm09	pdm09	pdm09	pdm09	pdm09	pdm09	pdm09	P
A/swine/Scotland/24678/2011	Scotland	2011	pdm09	pdm09	pdm09	pdm09	pdm09	pdm09	pdm09	pdm09	P
A/swine/England/058879/2011	England	2011	pdm09	pdm09	pdm09	pdm09	pdm09	pdm09	Scot94	Scot94	Q
A/swine/England/34627/2012	England	2012	pdm09	pdm09	pdm09	pdm09	pdm09	pdm09	Scot94	Scot94	Q
A/swine/England/041118/2013	England	2013	pdm09	pdm09	pdm09	pdm09	pdm09	pdm09	Scot94	Scot94	Q
A/swine/England/9953/2012	England	2012	pdm09	pdm09	pdm09	pdm09	pdm09	pdm09	Scot94	Scot94	Q
A/swine/England/015904/2012	England	2012	pdm09	pdm09	pdm09	pdm09	pdm09	pdm09	Scot94	Scot94	Q
A/swine/England/7856/2012	England	2012	pdm09	pdm09	pdm09	pdm09	pdm09	pdm09	Scot94	Scot94	Q
A/swine/England/038712/2012	England	2012	pdm09	pdm09	pdm09	pdm09	pdm09	pdm09	Scot94	Scot94	Q
A/swine/England/101878/2010	England	2010	pdm09	pdm09	pdm09	pdm09	pdm09	pdm09	Scot94	Scot94	Q
A/swine/England/142162/2010	England	2010	pdm09	pdm09	pdm09	pdm09	pdm09	pdm09	Scot94	Scot94	Q
A/swine/England/1380/2011	England	2011	pdm09	pdm09	pdm09	pdm09	pdm09	pdm09	Scot94	Scot94	Q
A/swine/England/90276/2011	England	2011	pdm09	pdm09	pdm09	pdm09	pdm09	pdm09	Scot94	Scot94	Q

A/swine/England/11449/2011	England	2011	pdm09	pdm09	pdm09	pdm09	pdm09	pdm09	Scot94	Scot94	Q
A/swine/England/112340/2011	England	2011	pdm09	pdm09	pdm09	pdm09	pdm09	pdm09	Scot94	Scot94	Q
A/swine/England/6183/2012	England	2012	pdm09	pdm09	pdm09	pdm09	pdm09	pdm09	Scot94	Scot94	Q
A/swine/England/7800/2012	England	2012	pdm09	pdm09	pdm09	pdm09	pdm09	pdm09	Scot94	Scot94	Q
A/swine/England/30148/2012	England	2012	pdm09	pdm09	pdm09	pdm09	pdm09	pdm09	Scot94	Scot94	Q
A/swine/England/024924/2012	England	2012	pdm09	pdm09	pdm09	pdm09	pdm09	pdm09	Scot94	Scot94	Q
A/swine/England/016947/2013	England	2013	pdm09	pdm09	pdm09	pdm09	pdm09	pdm09	Scot94	Scot94	Q
A/swine/England/61470/2013	England	2013	pdm09	pdm09	pdm09	pdm09	pdm09	pdm09	Scot94	Scot94	Q
A/swine/England/61025/2013	England	2013	pdm09	pdm09	pdm09	pdm09	pdm09	pdm09	Scot94	Scot94	Q
A/swine/England/072512/2013	England	2013	pdm09	pdm09	pdm09	pdm09	pdm09	pdm09	Scot94	Scot94	Q
A/swine/England/063782/2013	England	2013	pdm09	pdm09	pdm09	pdm09	pdm09	pdm09	Scot94	Scot94	Q
A/swine/Netherlands/263/2011	Netherlands	2011	pdm09	pdm09	pdm09	pdm09	pdm09	pdm09	pdm09	Gent84	R
A/swine/Netherlands/Barger-Compascuum-CVI6324A/2012	Netherlands	2012	pdm09	pdm09	pdm09	pdm09	pdm09	pdm09	pdm09	Gent84	R
A/swine/England/041406/2013	England	2013	pdm09	pdm09	pdm09	pdm09	pdm09	pdm09	pdm09	EA	S
A/swine/England/072865/2013	England	2013	pdm09	pdm09	pdm09	pdm09	pdm09	pdm09	pdm09	EA	S
A/swine/Hungary/362/2011	Hungary	2011	pdm09	pdm09	pdm09	pdm09	pdm09	pdm09	pdm09	EA	S
A/swine/Denmark/10802-1/2012	Denmark	2012	pdm09	pdm09	pdm09	pdm09	pdm09	pdm09	EA	Gent84	T
A/swine/Netherlands/409/2012	Netherlands	2012	pdm09	pdm09	pdm09	pdm09	pdm09	pdm09	EA	Gent84	T
A/swine/Spain/29113/2012	Spain	2012	pdm09	pdm09	pdm09	pdm09	pdm09	pdm09	EA	EA	U
A/swine/Spain/32738/2012	Spain	2012	pdm09	pdm09	pdm09	pdm09	pdm09	pdm09	EA	EA	U
A/swine/Belgium/113/2013	Belgium	2013	pdm09	pdm09	pdm09	pdm09	pdm09	pdm09	Gent84	Gent84	V
A/swine/Belgium/110/2013	Belgium	2013					EA	EA			Z
A/swine/France/CotesdArmor-0282/2011	France	2011	EA	EA	EA	EA	EA	EA	EA		Z
A/swine/Italy/7842/2011	Italy	2011				EA	EA	EA		Gent84	Z
A/swine/Italy/303558/2011	Italy	2011				EA	EA	EA			Z
A/swine/Italy/13133/2012	Italy	2012				EA	EA	EA		Gent84	Z
A/swine/Italy/317906-1/2011	Italy	2011				EA	EA	EA		EA	Z
A/swine/Italy/303682/2011	Italy	2011					EA	EA			Z
A/swine/Italy/311316/2012	Italy	2012					EA	EA			Z
A/swine/Italy/218884-4/2012	Italy	2012					EA	EA			Z
A/swine/Italy/154140-1/2012	Italy	2012	EA			EA	EA	EA		EA	Z
A/swine/Poland/18919/2009	Poland	2009		EA	EA		EA	EA		Scot94	Z
A/swine/Poland/15817/2011	Poland	2011	EA	EA	EA						Z
**A/swine/Italy/218884-2/2012**	**Italy**	**2012**	EA	EA	EA	EA	EA	EA	EA	EA	A
**A/swine/Italy/50814/2012**	**Italy**	**2012**	EA	EA	EA	EA	EA	EA	EA	EA	A
**A/swine/Spain/01/2010**	**Spain**	**2010**	EA	EA	EA	EA	EA	EA	EA	EA	A
**A/swine/Spain/02/2010**	**Spain**	**2010**	EA	EA	EA	EA	EA	EA	EA	EA	A
**A/swine/Spain/06/2011**	**Spain**	**2011**	EA	EA	EA	EA	EA	EA	EA	EA	A
**A/swine/Spain/1/2009**	**Spain**	**2009**	EA	EA	EA	EA	EA	EA	EA	EA	A
**A/swine/Italy/187969/2013**	**Italy**	**2013**	EA	EA	EA	EA	EA	EA	Gent-84	Gent-84	B
**A/swine/Spain/11/2010**	**Spain**	**2010**	EA	EA	EA	EA	EA	EA	Gent-84	Gent-84	B
**A/swine/Spain/12/2010**	**Spain**	**2010**	EA	EA	EA	EA	EA	EA	Gent-84	Gent-84	B

A/swine/England/00003/2009	England	2009	EA	EA	EA	EA	EA	EA	Scot-94	Scot-94	C
A/swine/Cotes_dArmor/1174/2009	France	2009	EA	EA	EA	EA	EA	EA	Scot-94	Scot-94	C
A/swine/Spain/07/2011	Spain	2011	EA	EA	EA	EA	EA	EA	Scot-94	Scot-94	C
A/swine/Italy/58769/2010	Italy	2010	EA	EA	EA	EA	EA	EA	EA	Gent-84	D
A/swine/Sweden/1021/2009	Sweden	2009	EA	EA	EA	EA	EA	EA	EA	Gent-84	D
A/swine/Sweden/9706/2010	Sweden	2010	EA	EA	EA	EA	EA	EA	EA	Gent-84	D
A/swine/Italy/191985/2009	Italy	2009		EA					Scot-94	It-N2	F
A/swine/Italy/289700/2009	Italy	2009		EA					Scot-94	It-N2	F
A/swine/Italy/320546/2009	Italy	2009	EA	EA	EA	EA	EA	EA	Scot-94	It-N2	F
A/swine/Italy/81226/2009	Italy	2009	EA	EA	EA	EA	EA	EA	Scot-94	It-N2	F
A/swine/Spain/05/2011	Spain	2011	EA	EA	EA	EA	EA	EA	Scot-94	EA	H
A/swine/Spain/09/2010	Spain	2010	EA	EA	EA	EA	EA	EA	Scot-94	huH3N2	J
A/swine/Italy/278299_1/2012	Italy	2012	EA	EA	EA	EA	EA	EA	pdm09	EA	K
A/swine/Italy/73449/2013	Italy	2013	EA	EA	EA	EA	EA	EA	pdm09	EA	K
A/swine/Wuerusreuth/IDT10990/2009	Germany	2009	pdm09	EA	pdm09	pdm09	EA	EA	Gent-84	pdm09	O
A/swine/England/10/2010	England	2010	pdm09	pdm09	pdm09	pdm09	pdm09	pdm09	pdm09	pdm09	P
A/swine/England/1458/2010	England	2010	pdm09	pdm09	pdm09	pdm09	pdm09	pdm09	pdm09	pdm09	P
A/swine/Heudorf-Messkirch/IDT14176/2011	Germany	2011	pdm09	pdm09	pdm09	pdm09	pdm09	pdm09	pdm09	pdm09	P
A/swine/Hungary/7380/2010	Hungary	2010	pdm09	pdm09	pdm09	pdm09	pdm09	pdm09	pdm09	pdm09	P
A/swine/Italy/225349-2/2012	Italy	2012	pdm09	pdm09	pdm09	pdm09	pdm09	pdm09	pdm09	pdm09	P
A/swine/Italy/287785/2012	Italy	2012	pdm09	pdm09	pdm09	pdm09	pdm09	pdm09	pdm09	pdm09	P
A/swine/Italy/290271/2009	Italy	2009	pdm09	pdm09	pdm09	pdm09	pdm09	pdm09	pdm09	pdm09	P
A/swine/Italy/325451/2011	Italy	2011	pdm09	pdm09	pdm09	pdm09	pdm09	pdm09	pdm09	pdm09	P
A/swine/Italy/85429/2009	Italy	2009	pdm09	pdm09	pdm09	pdm09	pdm09	pdm09	pdm09	pdm09	P
A/swine/Norway/02_11342/2009	Norway	2009	pdm09	pdm09	pdm09	pdm09	pdm09	pdm09	pdm09	pdm09	P
A/swine/Spain/04/2011	Spain	2011	pdm09	pdm09	pdm09	pdm09	pdm09	pdm09	pdm09	pdm09	P
A/swine/Albersloh/IDT14635-1/2012	Germany	2012	pdm09	pdm09	pdm09	pdm09	pdm09	pdm09	pdm09	Gent-84	R
A/swine/Duelmen/IDT14128/2011	Germany	2011	pdm09	pdm09	pdm09	pdm09	pdm09	pdm09	pdm09	Gent-84	R
A/swine/Freren/IDT13837-1/2011	Germany	2011	pdm09	pdm09	pdm09	pdm09	pdm09	pdm09	pdm09	Gent-84	R
A/swine/Garrel/IDT14891/2012	Germany	2012	pdm09	pdm09	pdm09	pdm09	pdm09	pdm09	pdm09	Gent-84	R
A/swine/Moeglingen/IDT14859/2012	Germany	2012	pdm09	pdm09	pdm09	pdm09	pdm09	pdm09	pdm09	Gent-84	R
A/swine/Papenburg/IDT12653/2010	Germany	2010	pdm09	pdm09	pdm09	pdm09	pdm09	pdm09	pdm09	Gent-84	R
A/swine/Saerbeck/IDT13755/2011	Germany	2011	pdm09	pdm09	pdm09	pdm09	pdm09	pdm09	pdm09	Gent-84	R
A/swine/Tornitz/IDT14598/2012	Germany	2012	pdm09	pdm09	pdm09	pdm09	pdm09	pdm09	pdm09	Gent-84	R
A/swine/Warendorf/IDT15018/2012	Germany	2012	pdm09	pdm09	pdm09	pdm09	pdm09	pdm09	pdm09	Gent-84	R
A/swine/Wettringen/IDT13795/2011	Germany	2011	pdm09	pdm09	pdm09	pdm09	pdm09	pdm09	pdm09	Gent-84	R
A/swine/Italy/116114/2010	Italy	2010	pdm09	pdm09	pdm09	pdm09	pdm09	pdm09	pdm09	Gent-84	R
A/swine/Garrel/IDT14844/2012	Germany	2012	pdm09	pdm09	pdm09	pdm09	pdm09	pdm09	pdm09	huH3N2	W

Supplementary Table 1. Overview of genome completion for the isolates used in this study. Strains isolated by the ESNIP3 consortium and analysed in this study are shown in regular font, while those obtained from the Influenza Virus Resource are shown in bold. For each isolate, the genome is separated into the internal gene cassette (PB2, PB1, PA, NP, MP, NS) and the external gene segments (HA and NA). The lineage-of-origin for each isolate's segment is given: EA = Eurasian 'avian-like' $H1_{av}N1$; Gent84 = A/swine/Gent/1/1984-derived H3N2; Scot94 = A/swine/Scotland/410440/1994-derived $H1_{hu}N2$; It-N2 = A/swine/Italy/4675/2003-derived N2; huH3N2 = human seasonal-like N2; pdm09 = A(H1N1)pdm09. The complete genotype code is as specified in Figure 1.
